# DPAGT1—Perspective as an Anticancer Drug Target

**DOI:** 10.3390/molecules30204049

**Published:** 2025-10-11

**Authors:** Michio Kurosu, Katsuhiko Mitachi

**Affiliations:** Department of Pharmaceutical Sciences, College of Pharmacy, University of Tennessee Health Science Center, 881 Madison Avenue, Memphis, TN 38163, USA

**Keywords:** DPAGT1, *N*-Glycan biosynthesis, anticancer drug target, DPAGT1 inhibitors, muraymycin A1, tunicamycins, capuramycin

## Abstract

Tunicamycins trigger endoplasmic reticulum (ER) stress by inhibiting DPAGT1 (dolichyl-phosphate *N*-acetylglucosamine-phosphotransferase 1): the rate-limiting enzyme that initiates *N*-glycan biosynthesis. Aberrant *N*-glycan branching is a hallmark of many solid tumors, and distinct cancer-associated *N*-glycan structures have been identified. Evidence shows that tunicamycins suppress key oncogenic processes, including proliferation, apoptosis resistance, metastasis, and angiogenesis. Yet their high systemic toxicity and lack of selectivity have precluded therapeutic application, and the structural complexity of tunicamycins has hindered chemical modification to mitigate these liabilities. No clinically translatable antitumor efficacy has been demonstrated in animal models. This review underscores the emergence of DPAGT1 as a novel and tractable anticancer target, outlining milestones in the discovery of selective inhibitors and their potential to transform cancer therapy. We discuss how advances in DPAGT1 inhibitor design may overcome limitations of tunicamycins and pave the way toward glycosylation-targeted oncology therapeutics.

## 1. Introduction

**Figure 1:**
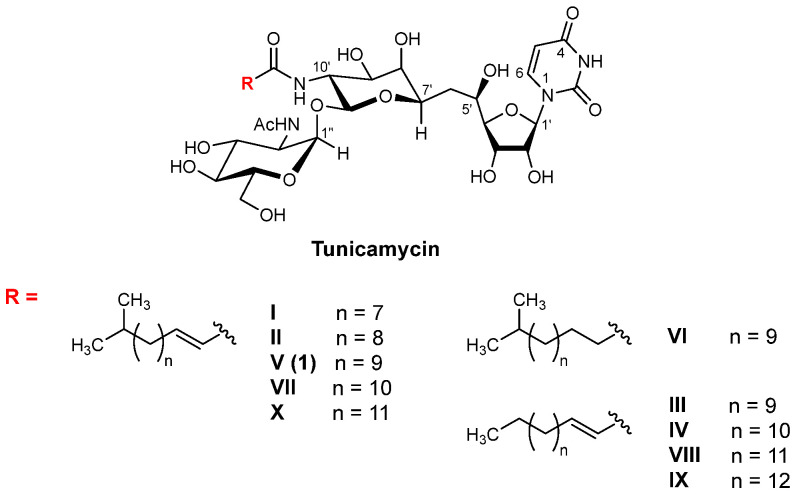
Structures of the tunicamycins.

Gene expressions of *N*- and *O*-glycan-associated glycoconjugates play important roles in carcinogenesis [1,2,3,4]. The structure and expression changes of glycoconjugates in cancers are known biological processes [5,6,7]. Aberrant glycosylation is a broadly recognized hallmark of cancer. DPAGT1 (dolichyl-phosphate *N*-acetylglucosamine-phosphotransferase 1) is a membrane-associated protein that catalyzes the first and rate-limiting step of *N*-glycan biosynthesis [2,8,9]. The correlation between the inhibition of DPAGT1 function and ER (endoplasmic reticulum)-stress responses followed by apoptosis has been studied using tunicamycins as non-selective DPAGT1 inhibitors of natural products [10,11,12,13,14,15,16,17]. Among tunicamycins, tunicamycin V (TM-V) is a major component that has been applied in cancer biology research [18,19,20]. TM-V is a moderate DPAGT1 inhibitor with a very narrow selective index (the toxic concentration/effective bioactive concentration) (Figure 1) [10,21]. At lower concentrations, TM-V inhibits multiple signaling pathways, eventually leading to apoptosis [22,23,24,25]. At higher concentrations, TM-V causes the death of mammalian cells via necroptosis and/or pyroptosis in a non-selective manner [10,21,26,27]. Overexpression of DPAGT1 has been reported in many solid cancers, including thyroid, lung, liver, carcinoid, pancreatic, head and neck, stomach, prostate, urothelial, testis, endometrial, breast, cervical, ovarian, skin, melanoma, and bladder cancers. Therefore, disruption of DPAGT1 function or suppression of DPAGT1 gene expression may result in unprecedented antiproliferative activity [28,29]. TM-V contains (1) two amide groups that are difficult to selectively deacylate and (2) an acid-labile 11′-β-1″-α-trehalose-type disaccharide linkage. Due to these structural complexities, chemical modifications aimed at mitigating the adverse effects of tunicamycins are particularly challenging [10]. Total chemical syntheses of tunicamycin V have been reported by several groups; however, their syntheses highlight new synthetic strategies, demonstrating the milligram quality of the synthesis [30,31,32,33,34]. We established highly efficient syntheses of nucleoside antibiotics that can be amenable to structure–activity relationship (SAR) studies [10,21,35,36]. This article summarizes new knowledge of DPAGT1 as an anticancer drug target obtained through our efforts on the discovery of selective DPAGT1 inhibitor molecules.

## 2. DPAGT1 and Its Inhibitor Molecules

**Figure 2:**
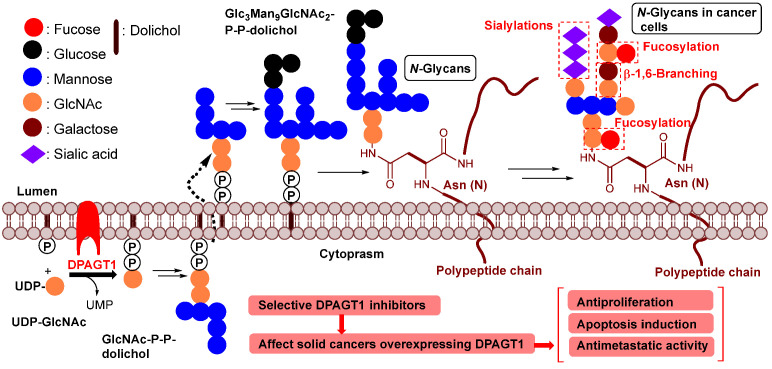
The process of *N*-linked glycosylation in mammalian cells. Typical modifications of *N*-glycans observed in cancer cells. DPAGT1 is overexpressed in many solid cancers.

DPAGT1 catalyzes the transformation from UDP-GlcNAc to GlcNAc-P-P-dolichol in the dolichol-linked oligosaccharide pathway, which is involved in *N*-glycan biosynthesis (Figure 2). Generated GlcNAc-P-P-dolichol in the ER is converted to the oligo-saccharide precursors by sequential enzymatic reactions, which are transferred to the Asn residues of polypeptides.

*N*-Glycans are further modified via serial glycosylations, yielding a wide variety of glycoprotein structures. Increases in β1,6-branchings, sialylations, and fucosylations are observed in *N*-glycans of cancer cells [2]. Specific *N*-glycan glycoconjugates have been identified in over 50% of cancerous cells [1,37,38,39]. Several glycosyltransferases associated with *N*-glycosylations are over-expressed in cancer cells [40]. These are the promising anticancer drug targets. However, the substrate-binding domains of glycosyltransferases are very hydrophilic [2]. Thus, it is considered one of the challenging medicinal chemistry programs to discover a strong glycosyltransferase inhibitor possessing an appropriate drug-like (hydrophobic) property. On the other hand, the DPAGT1 active sites have UDP-GlcNAc (hydrophilic) and dolichol-P (hydrophobic) binding sites [8,9]. TM-V is a known DPAGT1 inhibitor, and it has been applied to study unfolded protein response (UPR) at the endoplasmic reticulum (ER). However, TM-V shows promiscuous toxicity, limiting its applications in vitro at low concentrations and rarely in vivo via intratumoral administrations [41]. Discovery of alternative natural products or small molecules that have a strong DPAGT1 inhibitory activity with a wide therapeutic window will accelerate the study of the unfolded protein responses in cancers. The tunicamycins (Figure 1) were originally identified as narrow-spectrum antibiotics that exert their antibacterial activities by targeting translocase I (MraY/MurX) in peptidoglycan biosynthesis [10,18,19,20,35]. We thought that a new DPAGT1 inhibitor could be identified from the class of MraY inhibitors of nucleoside antibiotics represented by caprazamycin [42], muraymycins [43], liposidomycin [44], capuramycin [45], and their congeners [46,47,48].

## 3. Discovery of a New DPAGT1 Inhibitor of Natural Product

Aside from tunicamycins, commercially accessible natural-product MraY inhibitors are unavailable. However, the inherent cytotoxicity of tunicamycins as broad-spectrum phosphotransferase inhibitors, coupled with their undefined mechanisms, severely constrains their utility in cancer biology. Since the discovery of muraymycin A1 (MA1), the focus of MA1 has been its antibacterial activity [49,50,51]. However, cytotoxicity and DPAGT1 inhibitory activity of MA1 have not been examined. Insights from the DPAGT1–tunicamycin co-crystal structures [8,9] suggest that MA1 has the potential to disrupt DPAGT1 activity. To characterize MA1, we developed an efficient total synthesis, producing sufficient quantities for biological assays [52]. In reference to TM-V, MA1 was evaluated for its DPAGT1 enzyme inhibitory activity, antifungal activity, and cytotoxicity against both cancer and normal (healthy) cells (Table 1) [53]. We found that MA1 exhibits DPAGT1 enzyme inhibitory activity with an IC_50_ value more than eight times lower than that of TM-V in our assays. TM-V potently inhibited fungal growth at low concentrations (<1.0 µg/mL), whereas MA1 showed no detectable antifungal activity even at much higher concentrations (MIC > 35 µg/mL). Although *N*-glycosylation has been implicated in fungal pathogenicity [54,55], accumulated evidence shows that the inhibition of the fungal DPAGT1 homolog does not impair growth in culture. These findings suggest that the antifungal activity of TM-V reported in Table 1 arises from off-target mechanisms rather than direct N-glycosylation inhibition. Similarly, TM-V inhibited both cancerous and non-cancerous mammalian cells at low concentrations (<1.0 µM), exhibiting minimal selectivity with an average selectivity index (IC_50_ for cancer cells/IC_50_ for normal cells) of 1.25. The antiproliferative effects of MA1, summarized in Table 1, indicate that MA1 is non-toxic to normal cells (IC_50_ > 35 µM) but inhibits the growth of DPAGT1-overexpressing solid cancer cells. MA1 displayed cytotoxicity against high DPAGT1-expressing pancreatic, prostate, breast, cervical, ovarian, and melanoma cancer cells, whereas it was ineffective against low-DPAGT1-expressing cells, such as lymphoma cancers [53]. Healthy cells (MCF10A, HPNE, and Vero) listed in Table 1 were not affected by MA1. These assay results suggest the hypothesis that selective DPAGT1 inhibitors may serve as promising anticancer agents for the treatment of solid tumors that overexpress DPAGT1 [56]. The lack of toxicity of MA1 toward healthy cells represents the most significant discovery in the history of establishing DPAGT1 as an anticancer drug target. TM-V exerts rapid antiproliferative effects within 24–48 h, as reflected by the IC_50_ values at 48 h (Table 1). In contrast, MA1 and the other selective DPAGT1 inhibitors developed in our laboratory are slow-acting anticancer agents that require 72–96 h to exhibit antiproliferative activity, with corresponding IC_50_ values reported at 96 h (Table 1).

Because DPAGT1 is markedly overexpressed in the majority of solid tumor tissues compared with their healthy counterparts, MA1 stands out as a compelling lead compound. By selectively targeting DPAGT1, MA1 has the potential to become the first-in-class therapeutic inhibitor of this enzyme, thereby impairing *N*-glycan biosynthesis in cancer cells while minimizing adverse effects on normal tissues. Such a mechanism positions MA1 as a promising candidate for the development of a novel and highly selective anticancer therapy.

## 4. Muraymycin A1 Induces G_2_/M Cell Cycle Arrest and Apoptosis

**Figure 3:**
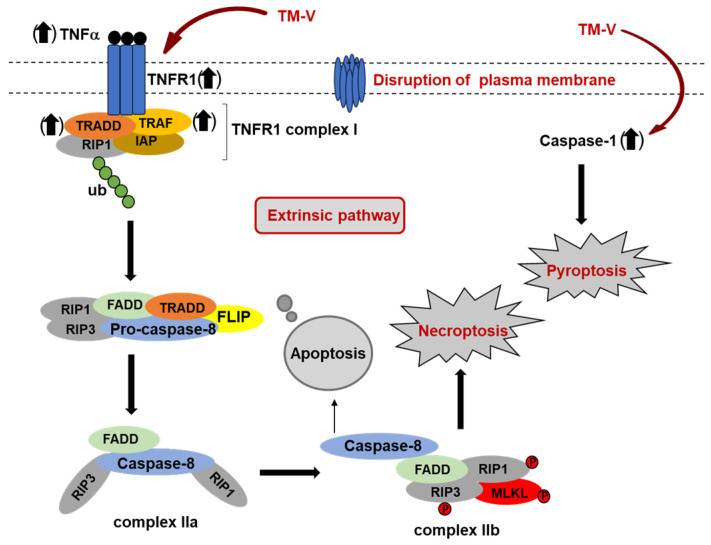
Mechanisms of the cell death of MDA-MB-231 treated with TM-V (major pathways).

The accumulation of unfolded proteins in the endoplasmic reticulum (ER) activates the unfolded protein response (UPR), a stress-signaling pathway that modulates transcription and translation (through effectors such as PERK, IRE1, and ATF6), ultimately inducing cell cycle arrest and subsequent cell death [57,58]. It is widely accepted that tunicamycins inhibit *N*-glycan biosynthesis, leading to cell cycle arrest at the G_0_/G_1_ phase in human cells [59,60,61,62,63]. The cell death mechanisms associated with tunicamycin treatment involve pathways beyond the UPR, and the molecular basis for tunicamycin-induced cell cycle arrest remains largely unknown. TM-V has a very narrow therapeutic window, inhibiting the growth of mammalian cells within 24–48 h at concentrations of 0.1–1.0 µM (Table 1). TM-V–induced cell death can occur through apoptosis, necroptosis, or a combination of both, depending on the treatment concentrations [64,65,66,67]. Cell cycle analyses by flow cytometry in a series of breast and pancreatic cancer cell lines revealed that MA1 induces arrest at the G_2_/M phase, whereas TM-V causes arrest at the G_0_/G_1_ phase, consistent with previously reported data [68,69]. Apoptosis and cell death mechanisms in MDA-MB-231 and AsPC-1 cells were assessed following treatment with TM-V (1.0 µM, 24 h) using annexin V and 7-aminoactinomycin D (7-AAD) staining. The analysis demonstrated that TM-V induces late-stage apoptosis, as evidenced by an increase in annexin V-positive/7-AAD-positive cells (with a significant rise in 7-AAD-positive cells), and also promotes cell death through necrotic and/or pyroptotic pathways, as indicated by SYTOX™-positive cells. These results suggest that TM-V triggers multiple forms of cell death in both breast and pancreatic cancer cell lines, highlighting its potent cytotoxic effects. We extensively investigated the cell death mechanisms in MDA-MB-231 cells treated with TM-V. TM-V does not trigger the release of cytochrome c from mitochondria into the cytoplasm, indicating that the intrinsic apoptotic pathway is not engaged. While the hallmark effect of DPAGT1 inhibitors on the inner mitochondrial membrane is a pronounced reduction in cardiolipin (CL) expression, TM-V treatment (1.0 µM, 24 h) did not alter CL levels much. These findings suggest that TM-V induces cell death through a mechanism independent of both cytochrome *c*-mediated apoptosis and cardiolipin depletion. Comprehensive analyses of mRNA and protein expression in TM-V–treated MDA-MB-231 cells demonstrated marked dysregulation of key mediators within the TNFR1 signaling cascade [70], culminating in necroptotic cell death and caspase-1 activation [71], which in turn facilitated the secretion of IL-1β and IL-18 [72,73], thereby triggering pyroptosis (Figure 3). Apoptosis triggered by the accumulation of misfolded proteins in the ER appears to represent only a minor intrinsic pathway in tunicamycin–treated cells, and the narrow therapeutic window of TM-V complicates the precise delineation of its apoptotic mechanisms.

**Figure 4:**
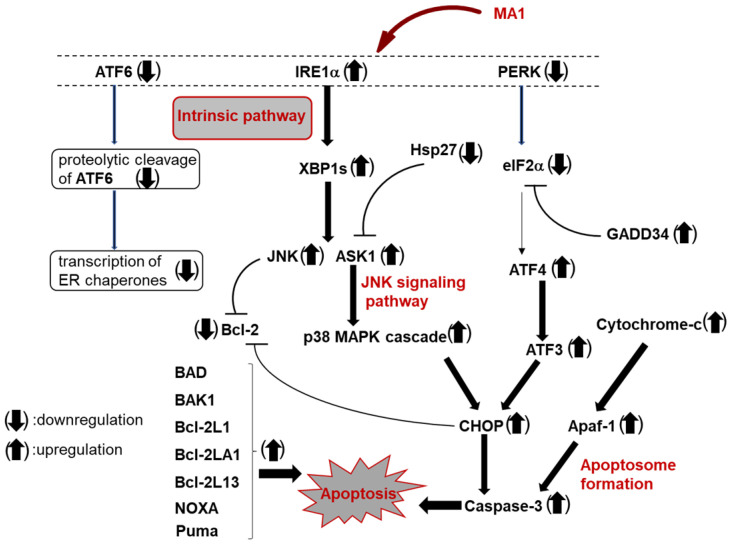
Induction of apoptosis in MDA-MB-231 treated with MA1.

In contrast, MDA-MB-231 cells treated with MA1 (0.2–1.0 µM for 48 h) exhibited a significant increase in apoptotic populations, as indicated by annexin V-positive staining relative to the untreated control. Mechanistic analyses demonstrated that MA1 induces the intrinsic (mitochondria-dependent) apoptotic pathway [74]. This was confirmed by multiple characteristic hallmarks of early apoptosis, including extensive DNA fragmentation, release of cytochrome *c* from mitochondria into the cytosol, and a pronounced reduction in cardiolipin (CL) expression, which is essential for maintaining inner mitochondrial membrane integrity [75,76]. Moreover, MA1 treatment led to activation and overexpression of executioner and initiator caspases (Caspase-3, -7, and -9), accompanied by the externalization of phosphatidylserine (PS) to the outer leaflet of the plasma membrane, a key feature of early apoptotic signaling [77,78]. Importantly, none of these apoptotic signatures were detected in MDA-MB-231 cells exposed to TM-V at the same concentration, underlining the distinct mechanisms of cell death triggered by MA1 versus TM-V. MA1 treatment of MDA-MB-231 cells increased the expression of BiP/GRP78, a central regulator of the UPR [79,80]. Under conditions of excessive ER stress, the UPR is mediated through three major signaling pathways: activating transcription factor 6 (ATF6), inositol-requiring enzyme 1α (IRE1α), and PKR-like endoplasmic reticulum kinase (PERK). MA1 upregulates the IRE1α pathway while downregulating ATF6 and PERK signaling (Figure 4). These alterations ultimately resulted in the overexpression of CHOP [81], activation of Caspase-3, and altered expression of BCL-2 family proteins [82,83], collectively driving the cells toward apoptosis.

MA1, unlike the promiscuously cytotoxic TM-V, is a stronger DPAGT1 inhibitor that selectively targets cancer cells without harming healthy cells at concentrations higher than 35 μM. Its distinct mechanisms of inducing cell death overturn the prevailing notion that DPAGT1 is an undruggable target in cancer therapy [2,10,21,52,56].

The antimigration (antimetastatic) activity of tunicamycins has been previously investigated [10,84]. However, TM-V exhibits only marginal antimigration activity at concentrations below 1.0 µM, likely due to its relatively weak inhibition of DPAGT1, an enzyme considered critical for the antimetastatic effects of tunicamycins [10,56]. In contrast, MA1 demonstrates strong antimigratory activity in the tested cancer cell lines [52,53]. Table 2 presents the biochemical properties of MA1 relative to those of tunicamycin V (TM-V).

## 5. Discovery of Novel DPAGT1 Inhibitors Inspired by Muraymycin A1

**Figure 5:**
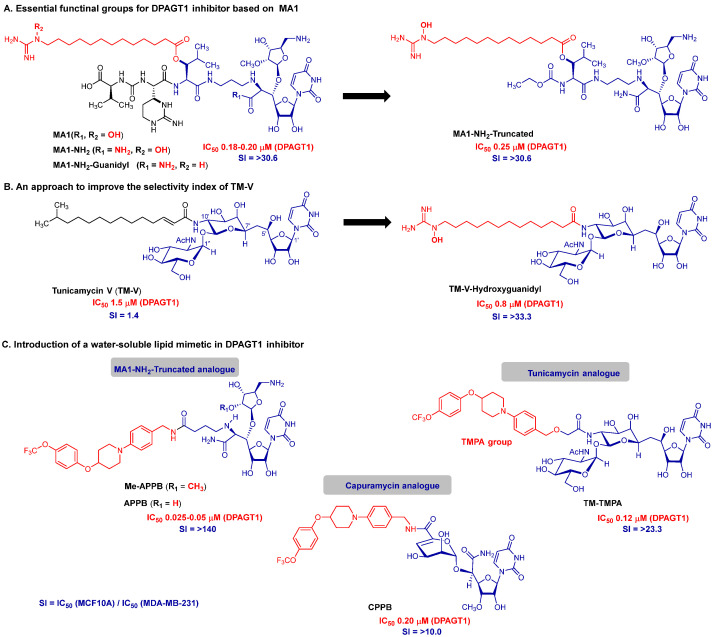
Novel DPAGT1 inhibitors discovered in the Kurosu lab.

The discovery of muraymycin A1 (MA1), a natural product that selectively inhibits human DPAGT1 without eliciting cytotoxicity in non-malignant cells, constitutes a landmark advance toward the development of mechanism-based anticancer agents targeting *N*-glycan biosynthesis for the treatment of metastatic solid cancers [53]. The synthesis of MA1 requires a highly complex strategy, which was accomplished for the first time by Dr. Kurosu and co-workers [52]. To understand the minimal structural requirements, we synthesized a series of key MA1 analogs and evaluated their inhibitory activity against DPAGT1 and their antiproliferative effects. MA1-amide (MA1-NH_2_) can be synthesized in significantly fewer steps than the natural product, providing a more accessible route for structure–activity relationship (SAR) studies (Figure 5A) [53].

MA1-NH_2_ exhibits comparable DPAGT1 inhibitory and antiproliferative activity to MA1. Replacement of the ω-hydroxyguanidyl (NH_2_-C(=NH)-N(OH)-) group in the fatty acid moiety of MA1 with a guanidyl (-NH_2_-C(=NH)-NH-) group does not significantly alter its enzyme inhibition or cancer cell growth inhibition. An important finding is that the peptide portion (diaminourea group) of MA1 is not essential for biological activity; the truncated analog, MA1-NH_2_-Truncated, retains comparable DPAGT1 inhibitory and antiproliferative activity, therefore defining a minimal structural requirement and offering a synthetically more accessible scaffold. MraY inhibitors enhance their antimycobacterial activity when modified with longer hydrocarbon chains, likely due to stronger interactions with mycobacterial cell wall components. However, this increased hydrophobicity often disrupts mammalian cell membranes, leading to cytotoxicity. One possible mechanism underlying the cytotoxicity of TM-V may involve this effect. To address this, we replaced the native C15-lipid chain of TM-V with the ω-hydroxyguanidyl lipid present in MA1. The resulting analog, TM-V-Hydroxyguanidyl, exhibited enhanced DPAGT1 inhibitory activity while eliminating cytotoxicity in noncancerous cells (Figure 5B). Thus, TM-V-Hydroxyguanidyl analogs provide an ideal biological probe to revisit ER stress response studies that were previously based on the promiscuous TM-V.

Water solubility is a key determinant of whether a bioactive compound can be advanced as a pharmacological agent, as poor solubility often translates into low bioavailability, formulation challenges, and limited therapeutic utility [21]. This issue is particularly common among complex natural products, many of which are originally isolated from organic solvent extracts and display inherently low aqueous solubility. TM-V, MA1, and their slightly modified analogs are no exception to this limitation. For example, the water solubility of TM-V is less than 0.2 mg/mL, a value far below what is considered favorable for drug development, therefore necessitating structural modification to improve physicochemical properties [10]. Recognizing this limitation, our work has been directed toward the rational design of a new family of molecules that retain the potent DPAGT1 inhibitory activity of their parent scaffolds, while exhibiting improved pharmacological attributes. In particular, our efforts focus on enhancing aqueous solubility without compromising biological activity, aiming to deliver candidates with both favorable safety profiles and practical drug-like properties suitable for clinical development. APPB (aminouridyl phenoxypiperidinbenzylbutanamide) and its methyl analog, Me-APPB, exhibit dramatically improved water solubility (75–79 mg/mL), a property that distinguishes them from earlier DPAGT1 inhibitors (Figure 5C) [35,36]. Both compounds display comparable potency, effectively suppressing the proliferation of diverse solid cancers—including breast, pancreatic, hepatic, oral, and cervical tumors—that depend on DPAGT1 overexpression for growth (IC_50_ = 0.1–0.8 μM). Notably, APPB and Me-APPB demonstrate remarkable selectivity, with minimal to no cytotoxicity toward noncancerous cells (IC_50_ > 35 μM), highlighting their potential as safe and targeted anticancer agents. In parallel, two additional water-soluble inhibitors, TM-TMPA and CPPB (capuramycin phenoxypiperidinbenzimide), were developed from tunicamycin and capuramycin scaffolds, thereby broadening the chemotype diversity available for selective DPAGT1 inhibition [10,56]. Collectively, more than 35 water-soluble lipid mimetics were identified that engage the dolichol-P binding domain with high efficiency, providing a versatile platform for pharmacological optimization. Details of their in vivo efficacy and therapeutic potential will be reported separately.

## 6. Proof-of-Pharmacological Concept

Due to their poor tolerability, tunicamycins are difficult to evaluate in rodent models, as systemic administration often results in severe toxicity (LD_50_ < 1.0 mg/kg) [85,86]. Their extremely poor aqueous solubility further complicates formulation, restricting dosing to very low concentrations that limit systemic exposure. Consequently, in vivo applications have generally been restricted to rare intravenous or intraperitoneal administrations, and more commonly direct intratumoral injections to achieve localized exposure [41]. These limitations make it difficult to disentangle on-target effects from nonspecific cytotoxicity. Considering that TM-V exhibits only moderate DPAGT1 inhibitory activity, the tumor regressions reported in TM-V–treated models are unlikely to be explained solely by DPAGT1 inhibition but may instead reflect a combination of partial target engagement and off-target cytotoxic mechanisms.

In vivo studies of APPB provide compelling validation that DPAGT1 inhibition is a promising strategy for the treatment of metastatic solid tumors [21]. APPB exhibits a favorable pharmacokinetic profile, with a biological half-life of ~6 h (IV), and is well tolerated, showing no acute toxicity at 20 mg/kg (IV, multiple doses, mouse). Extended administration (10 mg/kg daily for 45 days) did not produce detectable alterations in serum or plasma chemistry, underscoring a benign safety profile. Functionally, APPB demonstrated potent antitumor activity across multiple xenografts and orthotopic models, including AsPC1 (human pancreatic ductal adenocarcinoma), KPC-1 (mouse PDAC), MDA-MB-231 (triple-negative breast cancer), and Her2-positive patient-derived xenografts. Daily intraperitoneal dosing (2.5–10 mg/kg) significantly suppressed tumor growth, with efficacy comparable to standard-of-care therapies—paclitaxel in TNBC, gemcitabine in PDAC—and notably superior to trastuzumab in Her2-positive breast cancers. Importantly, APPB suppressed the growth of trastuzumab-resistant Her2-positive tumors, a critical unmet need. Strikingly, even slow-growing tumors such as AsPC-1 achieved complete regression within 22–35 days of treatment initiation. Collectively, these results establish APPB as not only a proof-of-concept DPAGT1 inhibitor but also a potential therapeutic candidate with efficacy and tolerability rivaling existing chemotherapies, while acting through a distinct and complementary mechanism of action. While tumor shrinkage is an essential metric of efficacy, equally critical is the impact on cancer-associated signaling pathways, one of which is highlighted in the following section.

## 7. Advancing DPAGT1 Inhibitors Toward Basic to Translational Studies

Tunicamycins, which target glycosylation modifications in specific organisms, have been investigated across various biomedical fields as part of fundamental research. The TM-Hydroxyguanidyl analog (Figure 5B) suppresses TM-V’s membrane-disrupting activity, enhancing selective toxicity while retaining MraY/WecA inhibition and improving DPAGT1 targeting. As a key biochemical tool, it enables re-evaluation of prior tunicamycin data, with effects now attributable specifically to DPAGT1 inhibition. This compound will be made accessible to the scientific community through our laboratory. The lead compounds in Figure 5C are being optimized through extensive medicinal chemistry in our laboratory to yield a clinical candidate for HER2 antibody–resistant breast cancer, supported by the California-based biotech, Anviron.

In contrast to conventional chemotherapeutics, which primarily exert their effects by targeting rapidly dividing cells, DPAGT1 inhibitors act through a fundamentally different mechanism. They interfere with *N*-glycan biosynthesis, a process that becomes particularly critical in cancer cells where DPAGT1 is overexpressed to sustain tumor growth and progression. This distinction is important, as it suggests that DPAGT1 inhibition may circumvent many of the limitations associated with traditional cytotoxic agents, such as broad toxicity against normal proliferating cells [2]. The favorable safety profile observed for the identified DPAGT1 inhibitors further highlights the therapeutic promise of this strategy and underscores the novelty of the present work.

Of particular significance, our findings demonstrate that APPB can act synergistically with established anticancer agents. In HER2 antibody–resistant breast cancer cell lines (SkBR3 and BT474 Clone 5), combination treatment of APPB with trastuzumab produced enhanced anticancer activity beyond that of each agent alone.

**Figure 6:**
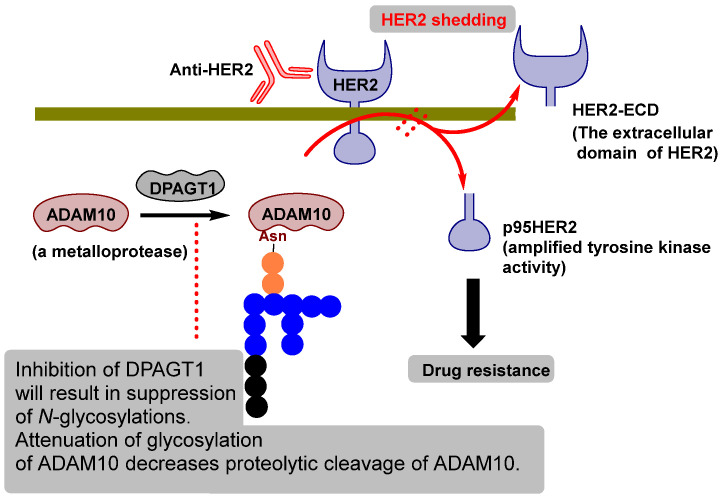
DPAGT1 inhibition suppresses HER2 shedding, restoring anti-HER2 activity.

These results suggest that DPAGT1 inhibition not only provides a discrete therapeutic axis but also holds the potential to resensitize-resistant tumors to standard-of-care agents. Together, these findings support the clinical potential of DPAGT1 inhibitors as a new class of targeted therapeutics, both as monotherapy and in rational drug combinations for refractory cancers. In this context, a particularly insightful study conducted by Yang et al. (Figure 6) sheds light on the role of ADAM10 in trastuzumab resistance [41]. Ectodomain shedding of membrane proteins is predominantly mediated by members of a disintegrin and metalloprotease (ADAM) family [87,88]. Aberrant activity of ADAM proteases has long been implicated in tumorigenesis and cancer progression [89,90], and more recently, elevated ADAM10 expression has been associated with poor clinical response to trastuzumab in HER2-positive breast cancers [91,92]. Importantly, ADAM10 has emerged as a principal determinant of HER2 ectodomain shedding [93]. In the study by Yang et al., DPAGT1-mediated *N*-glycosylation of ADAM10 was shown to be essential for its activation and stabilization. In the absence of this modification, ADAM10 was rapidly degraded within the intracellular compartments, therefore limiting its functional activity (Figure 6). By contrast, aberrant or excessive *N*-glycosylation stabilized ADAM10, facilitated its migration to the cell surface, and enhanced HER2 ectodomain cleavage, driving trastuzumab resistance [41]. Therapeutically, these findings were validated by treatment with TM-V, which inhibited ADAM10-mediated HER2 cleavage. Remarkably, this intervention re-sensitized trastuzumab-resistant tumors, leading to profound tumor regression in the trastuzumab resistant HER2+ breast cancer models. Collectively, this work highlights a mechanistic link between DPAGT1-mediated glycosylation and ADAM10 stabilization, offering a compelling rationale for targeting this pathway to overcome resistance to HER2-directed therapies. More than a decade of research has established a convincing case for the synergistic anticancer potential of DPAGT1 inhibitors. As illustrated in Table 3, the therapeutic possibilities of targeting *N*-glycan biosynthesis with safe and selective DPAGT1 inhibitors are vast, offering countless opportunities to explore novel strategies in cancer treatment [41,56,94,95,96,97,98,99,100,101,102,103].

## 8. Conclusions

Since their discovery, tunicamycins have been extensively investigated, with thousands of studies reporting their antitumor activity in both in vitro and in vivo models. However, the development of selective DPAGT1 inhibitors has introduced unprecedented precision, providing powerful biochemical tools to dissect DPAGT1-dependent mechanisms and reinterpret findings previously attributed to broadly acting tunicamycins. These selective inhibitors open up new opportunities for targeted therapeutic strategies as well as mechanistic studies in cancer biology.

Muraymycin A1 (MA1), a newly identified natural product inhibitor of DPAGT1, holds strong potential to improve human health and treat diseases associated with prolonged ER stress, including inflammation- and immune-related disorders, cardiovascular disease, metabolic syndromes, and cancer. In this work, we focus on the application of safer DPAGT1 inhibitors for cancer therapy. Through rational molecular design, natural products such as MA1, TM-V, and capuramycin can be engineered into selective DPAGT1 inhibitors. As noted above, tunicamycins have long served as important investigational tools in the study of diverse human diseases. The development and pharmacological validation of selective DPAGT1 inhibitors should now enable a more accurate re-examination of these biochemical processes, paving the way for therapeutic insights beyond those provided by tunicamycins.

## Figures and Tables

**Table 1:** DPAGT1 enzyme inhibitory, antifungal, and cytotoxicity activities of muraymycin A1 (MA1) in comparison to tunicamycin V (TM-V).

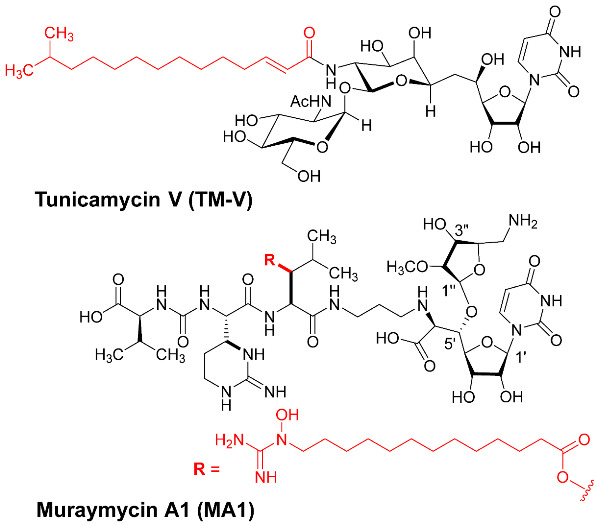			
Target	IC_50_ or MIC	TM-V	MA1
Enzyme	DPAGT1 (IC_50_)	1.5 μM	0.18 μM
Fungus	*C. albicans* 18M (MIC)	<0.24 μg/mL	>35 μg/mL
	*C. neoformans* NIH9hi90 (MIC)	0.24 μg/mL	>35 μg/mL
	*C. gatti* C17 (MIC)	<0.24 μg/mL	>35 μg/mL
	*A. fumigaus* ASFU-2263 (MIC)	0.96 μg/mL	>35 μg/mL
DPAGT1-high expression cancer cells (Breast cancers)	MDA-MB-231 (IC_50_)	0.17 μM (48 h)	0.98 μM (96 h)
	MDA-MB-453 (IC_50_)	0.16 μM (48 h)	0.85 μM (96 h)
	MDA-MB-468 (IC_50_)	0.25 μM (48 h)	0.85 μM (96 h)
	SkBr3 (IC_50_)	0.11 μM (48 h)	0.56 μM (96 h)
	BT-474 (IC_50_)	0.15 μM (48 h)	0.85 μM (96 h)
	HCC38 (IC_50_)	0.35 μM (48 h)	0.95 μM (96 h)
	MCF7 (IC_50_)	0.54 μM (48 h)	0.95 μM (96 h)
DPAGT1-low expression cancer cells	HCT116 (IC_50_)	0.50 μM (48 h)	>35 μM (96 h)
	HT-29 (IC_50_)	0.45 μM (48 h)	>35 μM (96 h)
	HepG2 (IC_50_)	0.98 μM (48 h)	>35 μM (96 h)
	Caco-2 (IC_50_)	0.55 μM (48 h)	>35 μM (96 h)
	HL-60 (IC_50_)	0.45 μM (48 h)	>35 μM (96 h)
	L1210 (IC_50_)	0.35 μM (48 h)	>35 μM (96 h)
Normal (healthy) cells	MCF10A (IC_50_)	0.25 μM (48 h)	>35 μM (96 h)
	HPNE (IC_50_)	0.15 μM (48 h)	>35 μM (96 h)
	Vero (IC_50_)	0.25 μM (48 h)	>35 μM (96 h)

**Table 2:** Biochemical property of muraymycin A1 (MA1) in comparison to tunicamycin V (TM-V).

	TM-V	MA1
DPAGT1 (IC_50_)	1.5 μM	0.18 μM
Cell cycle arrest	G_0_/G_1_	G_2_/M
Cell death	Apoptosis (+) (non-apoptotic cell death processes)	Apoptosis (+++)
Cytotoxicity rate	24−48 h (at 0.10−1.0 μM)	72−96 h (at 0.50−0.98 μM)
Selectivity Index (SI)	0.5−2.3	>30
Migration inhibition	+ (0.4−1.0 μM)	+++ (0.4−1.0 μM)

+: Weak, +++: Strong.

**Table 3:** Reported synergistic action of tunicamycins.

Cancer Type	Mechanisms of Action(s)	Synergies
Prostate cancer [94]	Disruption of mTORC1 pathway Induction of UPR apoptosis	Docetaxel
Breast cancer (Her2+) [41,95,96]	Prevent glycosylation of ADAM10 Regulation of Akt/NF-kB pathway Activate UPR apoptosis in high DPAGT1-expressing cells Induction of UPR apoptosis	Anti-Her2
Breast cancer (TNBC) [97,98,99,100]	Disruption of Wnt/β-catenin pathway Inhibition of EGRF (and PD-L1 expression) Inhibitions of PI3K Induction of UPR apoptosis Induction of GRP78 and CHOP/GADD153 Disruption of PD1/PD-L1 binding	Doxorubcin Anti-Her2
Lung (NSCLC) and Liver cancer [101,102]	Glycosylated PTX3 via Akt/NF-kB Induction of UPR apoptosis Inhibition of EGFR	Erlotinib Cisplatin
Pancreatic cancer [103]	Disruptions of Wnt/β-catenin/Snail pathways Inhibition of WYC Induction of UPR apoptosis	Paclitaxel Gemcitabine 5-FU
Pancreatic cancer [56]	Induction of UPR apoptosis Inhibition of Snail and enhancement of E-cadherin expression	Paclitaxel

## Data Availability

No new data were created or analyzed in this study.

## Abbreviations

The following abbreviations are used in this manuscript:

DPAGT1	Dolichyl-phosphate *N*-acetylglucosamine-phosphotransferase 1
ER	Endoplasmic reticulum
TM-V	Tunicamycin V
LD	Linear dichroism
UDP-GlcNAc	Uridine diphosphate *N*-acetylglucosamine
Asn	Asparagine
MraY	Phospho-MurNAc-pentapeptide translocase
WecA	*N*-Acetylglucosamine-1-phosphate transferase
*C. albicans*	*Candida albicans*
*C. neoformans*	*Cryptococcus neoformans*
*C. gattii*	*Cryptococcus gattii*
*A. fumigatus*	*Aspergillus fumigatus*
MDA-MB-231	Human triple negative breast cancer
MDA-MB-453	Human triple negative breast cancer
MDA-MB-468	Human triple negative breast cancer
SkBr3	HER2-overexpressing human breast cancer
BT-474	HER2+ human ductal breast carcinoma
HCC38	Triple-negative breast cancer
MCF7	A human breast adeno-carcinoma cell (ER+)
HCT116	Human colon cancer.
HT-29	Human colorectal adenocarcinoma
HepG2	Human hepatoma cell
Caco-2	Human colorectal adenocarcinoma cell
HL-60	Human leukemia cell
L1210	Mouse lymphocytic leukemia cell
MCF10A	Human mammary epithelial cell
HPNE	Human pancreatic ductal cell
Vero	African green monkey kidney cell.
MA1	Muraymycin A1
IC_50_	Half-maximal inhibitory concentration
MIC	Minimum inhibitory concentration
PERK	Protein kinase R (PKR)-like endoplasmic reticulum kinase
IRE1	Inositol-requiring enzyme 1
ATF6	Activating transcription factor 6
UPR	Unfolded protein response
7-AAD	7-Aminoactinomycin D
TNFR1	Tumor necrosis factor receptor 1
IL	Interleukin
CL	Cardiolipin
PS	Phosphatidylserine
BiP	Binding immunoglobulin protein
GRP78	78 kDa glucose-regulated protein
CHOP	C/EBP homologous protein (DNA damage-inducible transcript 3)
BCL-2	B-cell lymphoma 2 protein
APPB	Aminouridyl phenoxypiperidinbenzylbutanamide, a DPAGT1 inhibitor
Me-APPM	A methylated analog of APPB
CPPB	Capuramycin phenoxypiperidinbenzimide, a DPAGT1 inhibitor
TM-TMPA	A DPAGT1 inhibitor of tunicamycin analog
LD_50_	The median lethal dose
IV	Intravenous administration
KPC-1	Mouse pancreatic ductal adenocarcinoma
Her2	Human epidermal growth factor receptor 2
mTORC1	Mammalian target of rapamycin complex 1
ADAM10	Metalloproteinase domain-containing protein 10
UPR	The unfolded protein response
Akt	Protein kinase B
NF-kB	Nuclear factor kappa-light-chain-enhancer of activated B
Wnt	A proto-oncogene *Wnt-1* protein
β-catenin	A protein encoded by the *CTNNB1* gene
EGF	Epidermal growth factor
PD-L1	Programmed death-ligand 1
PI3K	Phosphoinositide 3-kinase
PTX3	Pentraxin-related protein, TNF-inducible gene 14 protein
NSCLC	Non-small cell lung cancer
WYC	A retinoic acid receptor agonist
5-FU	5-Fluorouracil
BT474 Clone 5	An epithelial-like cell line clone of ductal carcinoma breast cancer
